# The ERP and psychophysical changes related to facial emotion perception by expertise in Japanese hospitality, “OMOTENASHI”

**DOI:** 10.1038/s41598-022-11905-2

**Published:** 2022-06-14

**Authors:** Kensaku Miki, Yasuyuki Takeshima, Tetsuo Kida, Ryusuke Kakigi

**Affiliations:** 1 Department of Integrative Physiology, National Institute for Physiological Sciences, National Institutes of Natural Sciences, Okazaki, Aichi Japan; 2 Integrative Physiology, College of Nursing, Aichi Medical University, 1-1 Yazakokarimata, Nagakute, Aichi 480-1195 Japan; 3 School of Nursing, Japanese Red Cross Toyota College of Nursing, Toyota, Aichi Japan; 4 Institute for Developmental Research, Aichi Developmental Disability Center, Kaugai, Aichi Japan

**Keywords:** Neuroscience, Physiology, Psychology, Systems biology

## Abstract

We investigated the emotion perception process based on hospitality expertise. Forty subjects were divided into the OMOTENASHI group working at inns considered to represent the spirit of hospitality, OMOTENASHI in Japan, and CONTROL group without experience in the hospitality industry. We presented neutral, happy, and angry faces to investigate P100 and N170 by these faces, and psychophysical changes by the favor rating test to evaluate emotional perception. In the favor rating test, the score was significantly smaller (less favorable) in OMOTENASHI than in CONTROL. Regarding event-related potential components, the maximum amplitude of P100 was significantly larger for a neutral face at the right occipital electrode in OMOTENASHI than in CONTROL, and it was significantly larger for an angry face at both occipital electrodes in OMOTENASHI than in CONTROL. However, the peak latency and maximum amplitude of N170 were not significantly different between OMOTENASHI and CONTROL at both temporal electrodes for each emotion condition. Differences on the favor rating test and P100 in OMOTENASHI suggested that workers at inns may more quickly notice and be more sensitive to the facial emotion of guests due to hospitality training, and/or that hospitality expertise may increase attention to emotion by top-down and/or bottom-up processing.

## Introduction

In our daily lives, the information from the face, for example, sex, age, and familiarity, is important. Facial emotion, in particular, is essential for our social communication, because it reflects the state of mind of others, and humans can read and sympathize with their state.

In many psychological studies, models of face recognition have been hypothesized. Bruce and Young^1^ suggested a model in which faces are initially processed in a stage of structural encoding, which includes view-centered and expression-independent descriptions of faces. After this description, facial identification, facial speech, and emotional expression are processed in parallel and independently. Facial identification begins from structural encoding and arrives at a face recognition unit (FRU), where structural codes of familiar faces are stored.

Non-invasive neuroimaging methods were recently used to investigate the human perception and recognition systems. Event-related potentials (ERPs) using electroencephalography (EEG) are especially useful to investigate the time course of perception and recognition processes in humans because of the high temporal resolution. There have been many studies about face recognition using ERPs, and N170 is known as a major component specific to face recognition and is mainly elicited approximately 170 ms at both temporal electrodes after the presentation of a face^2,3^. The characteristics of N170 are as follows: (1) N170 is larger for faces than for animals, cars, or chairs. (2) N170 is larger for faces at the right temporal electrode than at the left. (3) N170 is smaller for upright faces than for inverted faces, i.e., face inversion effect^4^. (4) N170 is smaller for upright faces than for eyes.

P100 (P1) and N100 are also known as components related to the early process of visual perception in general, and begin 90–120 ms at occipital electrodes after luminance^2,5^. The Pl00 recorded was not sensitive to stimulus category, but rather to stimulation of luminance, luminance contrast, and size^5^, whereas N100 was larger for a scrambled face than for an unscrambled face^2^. In addition, P100 is affected by visual attention^6,7^. P100 is elicited involuntarily at the appearance of a visual stimulus when not attended, whereas its amplitude increases when a person allocates attention to the stimulus.

There have been several studies on facial emotion using ERPs^8–11^. Regarding the N170 component, a meta-analysis demonstrated that anger, fear, and happiness elicited the largest amplitudes of N170 with respect to neutral, non-expressive faces^11^, and another study systematized facial emotion effects as a function of different attention, and revealed that N170 emotion effects are most consistently found in passive viewing designs^12^. In addition, other studies reported the following characteristics about the alteration of N170 by emotion and naturalness: (1) It is independent of perceptual load, but regulated by the inter-stimulus interval (ISI)^13^. (2) The N170 emotional alteration is task-independent and unaffected by feature-based attention^14^. (3) Fearful faces and decreasing face naturalness elicited a substantially larger N170^15^. (4) N170 emotional alteration requires the processing of figural facial expression features^16^. (5) N170 emotional alteration depends mainly on configural information of faces, i.e., recognizing a face^17^. (6) N170 emotional alteration is unaffected by expression-specific spatial frequencies^18^.

As for P100, previous ERP studies revealed that it is also influenced by facial emotion. Dennis et al.^10^ found that the latency of P100 was shortened in response to a fearful face compared with a sad face, and P100 emotion and face effects are strongly influenced by low-level information in contrast to the N170 component^16,19^. Furthermore, the following characteristics about the alteration of P100 by emotion and naturalness were noted: (1) P100 emotional alteration is dependent of perceptual load, irrespective of the ISI^13^. (2) It appears strongly in perceptual tasks^13^. (3) Lower face naturalness results in a larger P100^15^. (4) P100 emotional alteration strongly depends on low-level visual information^16^. (5) Expression-specific spatial frequencies alter P100^18^.

The process of human perception and recognition is also affected by many factors such as age, expertise, and training. Many previous studies reported that ERPs related to facial emotion change with age. Batty and Taylor^20^ revealed that the sensitivity of N170 to emotions in adults appeared late at 14 to 15 years of age. Based on our previous study^21^, the areas of the brain involved in perceiving changes in facial emotion have not matured by 14 years of age, and we hypothesized that the developmental changes in the perception of facial emotional change are driven, in part, by different factors, such as skull growth and myelination in the visual cortex, and by perceptual and cognitive development.

In Japan, the expression of kind, warm, and sensitive hospitality is termed “OMOTENASHI”. Workers at Japanese hot spring spa inns are considered representatives of OMOTENASHI. This is the major reason for why Japanese prefer staying at such inns, and foreign travelers also enjoy not only hot spring spas, but also this expression of OMOTENASHI. In addition, workers at hot spring spa inns observe guests carefully, thus workers at hot spring spa inns can judge what kind of person guests are, how guests are, and whether guests are favorable to inn. However, no studies have investigated OMOTENASHI.

Therefore, we investigated OMOTENASHI by evaluating the processes of facial emotion perception. The main objective of this study was to assess whether the facial emotion perception is affected by the existence of expertise in Japanese hospitality (OMOTENASHI). In this study, we investigated the differences in P100 and N170 of ERPs, and psychophysical changes by an original test evaluating the characteristics of emotional perception between subjects who worked at hot spring spa inns and those without experience in the hospitality industry.

## Results

### Psychophysical study

The *stimulus condition* (F = 201.689, p < 0.05, partial η^2^ = 0.841) and *subject group* (F = 9.982, p < 0.05, partial η^2^ = 0.208) had significant effects on the score on the favor rating test. The score was significantly smaller in OMOTENASHI than in CONTROL (Table 1). The score for Neutral was significantly smaller in OMOTENASHI than in CONTROL by Welch's t test (p < 0.05) (Table 1).

**Table 1:** The score on favor rating tests for Neutral, Happy, and Angry stimulus conditions in OMOTENASHI and CONTROL.

Score	OMOTENASHI	CONTROL
Neutral	3.3 ± 1.1*	4.1 ± 1.1
Happy	5.8 ± 1.5	6.4 ± 0.8
Angry	1.3 ± 0.4	1.7 ± 0.9

Data are presented as the mean and standard deviation for each group.

*p < 0.05: comparison with CONTROL.

### P100

#### Waveform of P100

**Figure 1:**
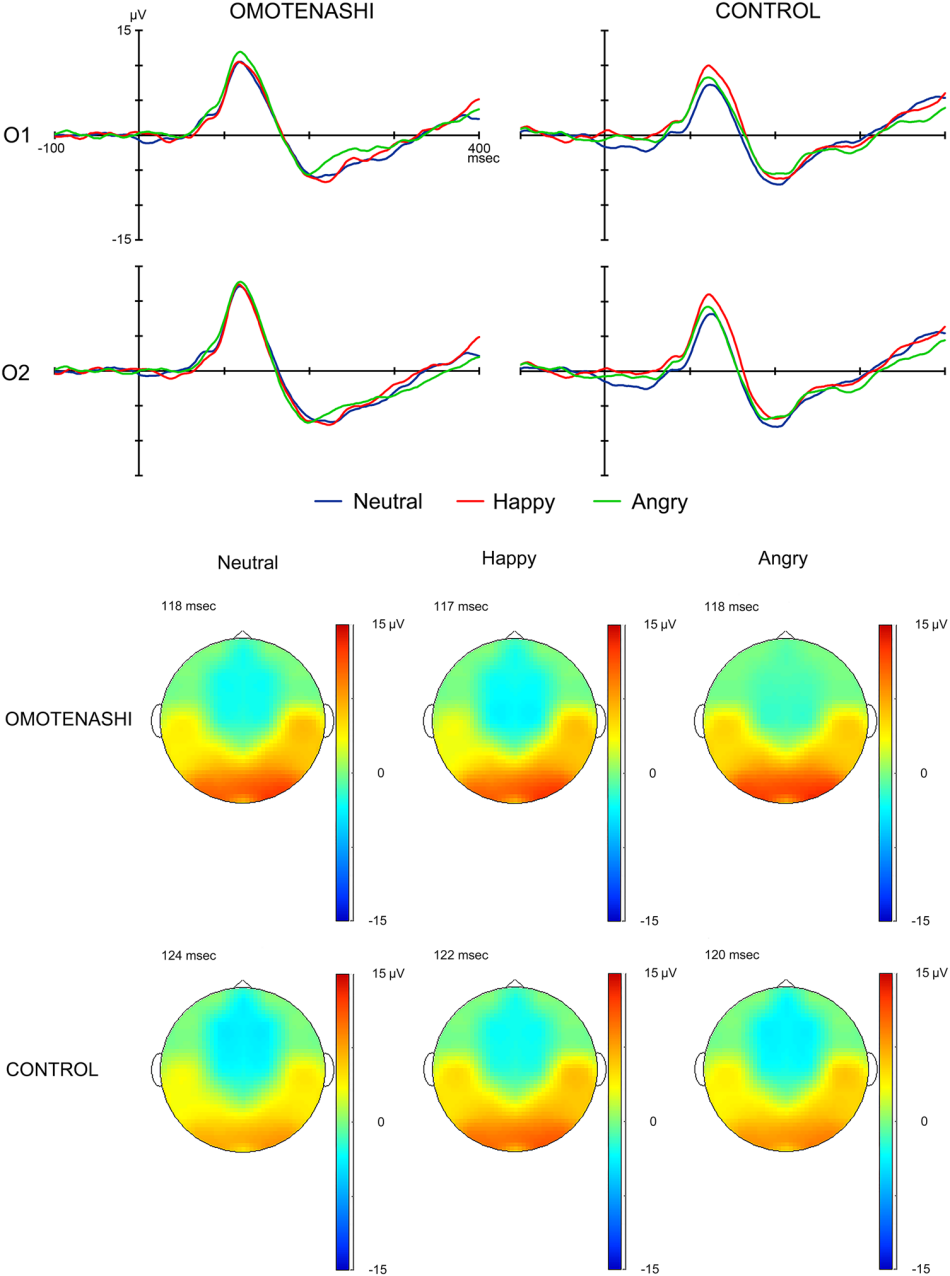
The grand-averaged waveforms of P100 at O1 and O2 of the left and right occipital areas (upper), and the topography map of P100 (lower) for Neutral, Happy, and Angry in OMOTENASHI and CONTROL.

The grand-averaged waveforms of P100 obtained for each subject group for all stimulus conditions (Neutral, Happy, and Angry) by the O1 (left) and O2 (right) electrodes are shown in Fig. 1. The mean (and standard deviation) values in the peak latency and maximum amplitude (baseline-to-peak) of P100 obtained for each group (OMOTENASHI or CONTROL) for each stimulus condition (Neutral, Happy, or Angry) by the O1 (left) and O2 (right) electrodes are shown in Tables 2 and 3.

**Table 2:** Peak latency of P100 at O1 (left) and O2 (right) for Neutral, Happy, and Angry stimulus conditions in OMOTENASHI and CONTROL.

Latency (ms)	OMOTENASHI		CONTROL	
	O1	O2	O1	O2
Neutral	128.0 ± 18.0	124.7 ± 12.7	130.7 ± 14.5	128.6 ± 11.7
Happy	126.3 ± 16.6	121.8 ± 12.7	129.2 ± 14.2	128.0 ± 12.3
Angry	124.0 ± 19.4	123.6 ± 14.1	126.9 ± 16.9	126.7 ± 13.6

Data are presented as the mean and standard deviation for each group.

**Table 3:** Maximum amplitude (baseline-to-peak) of P100 at O1 (left) and O2 (right) for Neutral, Happy, and Angry stimulus conditions in OMOTENASHI and CONTROL.

Amplitude (µV)	OMOTENASHI		CONTROL	
	O1	O2	O1	O2
Neutral	12.1 ± 5.5	13.7 ± 5.2*	9.2 ± 3.6	10.2 ± 4.5
Happy	11.8 ± 4.7	13.2 ± 5.4	10.8 ± 3.3	11.7 ± 3.1
Angry	13.6 ± 5.2**	14.2 ± 5.3**	9.6 ± 3.6	10.1 ± 3.8

Data are presented as the mean and standard deviation for each group.

*p < 0.05: comparison with CONTROL.

**p < 0.01: comparison with CONTROL.

#### Peak latency of P100

The *electrode* (F = 4.606, p < 0.05, partial η^2^ = 0.108) had a significant effect on the peak latency of P100. The peak latency of P100 was shorter at the O2 electrode (right) than at the O1 electrode (left). However, *stimulus condition* (F = 1.410, p > 0.05, partial η^2^ = 0.036) and *subject group* (F = 0.799, p > 0.05, partial η^2^ = 0.021) did not have significant effects on P100 latency.

We investigated the differences in the peak latency of P100 among the *stimulus condition* in each subject group at each electrode using post hoc analysis. *Stimulus* *condition* and *electrode* did not have significant effects on the peak latency of P100 in either subject group. In addition, we investigated the differences in the latency among *subject group* for each stimulus at each electrode. *Subject group* did not have a significant effect on the peak latency of P100.

#### Maximum amplitude of P100

The *subject group* (F = 6.662, p < 0.05, partial η^2^ = 0.149) and *electrode* (F = 4.110, p < 0.05, partial η^2^ = 0.098) had significant effects on the maximum amplitude of P100. The maximum amplitude of P100 was significantly larger in OMOTENASHI than in CONTROL. However, the effect of *stimulus condition* (F = 0.491, p > 0.05, partial η^2^ = 0.013) on the maximum amplitude of P100 was not significant.

We investigated the differences in the maximum amplitude of P100 among the *stimulus condition* in each group at each electrode using post hoc analysis. Among the *stimulus condition*, there were no significant effects on the maximum amplitude of P100 in each group at each electrode. In addition, we investigated the differences in the maximum amplitude of P100 among *subject group* for each stimulus at each electrode by Welch's t test. After the presentation of Neutral, the maximum amplitude of P100 was significantly larger in OMOTENASHI than in CONTROL at the O2 electrode (right). In addition, after the presentation of Angry, the maximum amplitude of P100 was significantly larger in OMOTENASHI at O1 (left) and O2 (right) electrodes than in CONTROL.

#### Topographical map of P100

The topographical maps of P100 when the P100 amplitude in grand-averaged waveforms was largest at the O2 electrode (right) in each subject group for all stimulus conditions (Neutral, Happy, and Angry) are shown in Fig. 1. The amplitude at the bilateral occipital areas was larger for Neutral and Angry in the OMOTENASHI than in the CONTROL, and was larger for Happy than Neutral or Angry in the CONTROL.

### N170

#### Waveform of N170

**Figure 2:**
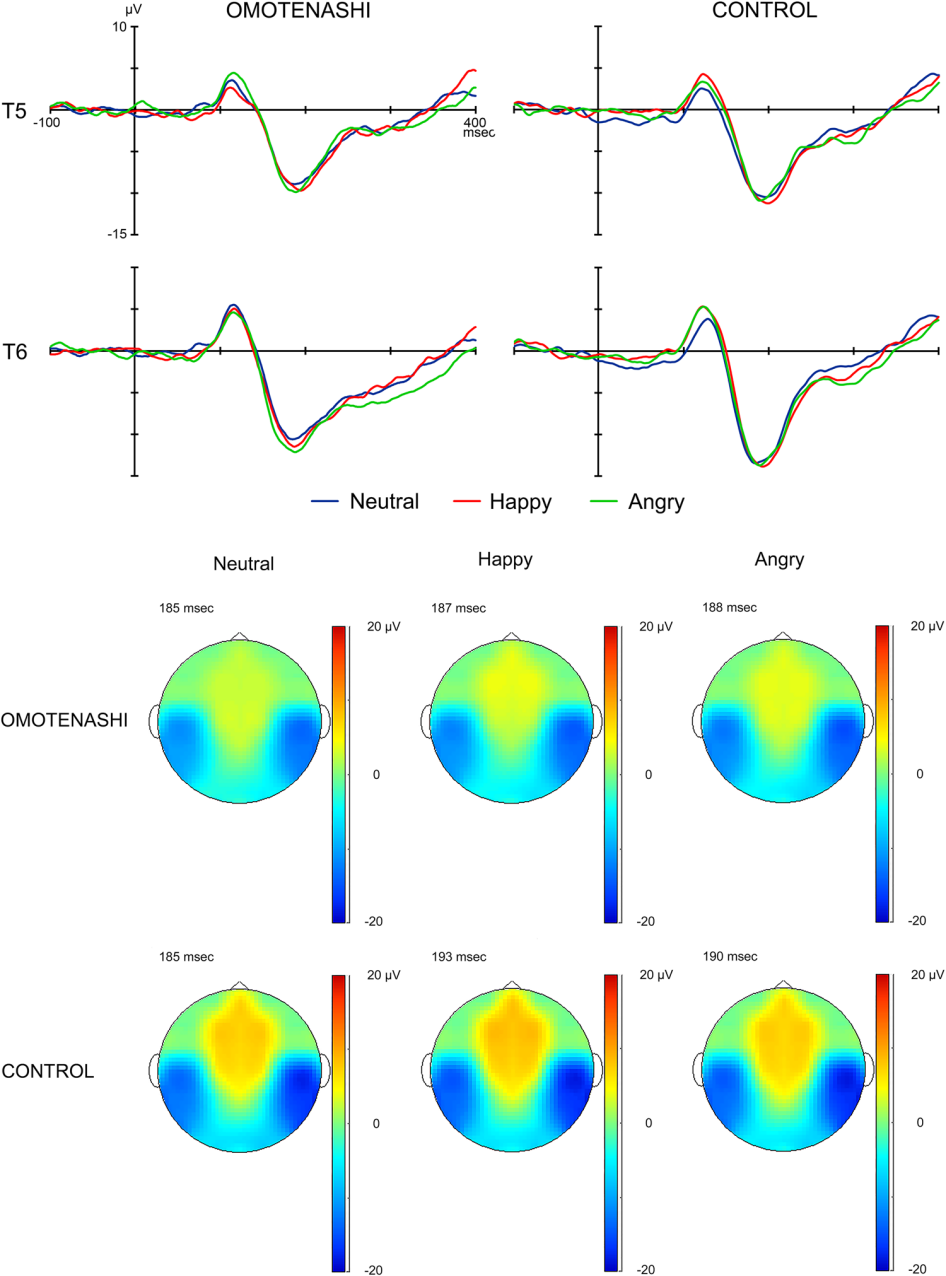
The grand-averaged waveforms of N170 at T5 and T6 of the left and right temporal areas (upper), and the topography map of N170 (lower) for Neutral, Happy, and Angry in OMOTENASHI and CONTROL.

The grand-averaged waveforms of N170 recorded for each subject group in each stimulus condition (Neutral, Happy, or Angry) by the T5 (left) and T6 (right) electrodes are shown in Fig. 2. The mean (and standard deviation) values in the peak latency and maximum amplitude (baseline-to-peak) of N170 obtained for each group (OMOTENASHI or CONTROL) for each stimulus condition (Neutral, Happy, or Angry) by the T5 (left) and T6 (right) electrodes are shown in Tables 4 and 5.

**Table 4:** Peak latency of N170 at T5 (left) and T6 (right) for Neutral, Happy, and Angry stimulus conditions in OMOTENASHI and CONTROL.

Latency (ms)	OMOTENASHI		CONTROL	
	T5	T6	T5	T6
Neutral	189.8 ± 28.8	192.9 ± 33.7	190.5 ± 19.4	187.3 ± 19.1
Happy	191.8 ± 20.1	197.5 ± 31.8	193.3 ± 21.0	191.7 ± 16.6
Angry	189.9 ± 17.2	195.9 ± 33.7	190.4 ± 19.7	189.9 ± 17.7

Data are presented as the mean and standard deviation for each group.

**Table 5:** Maximum amplitude of N170 at T5 (left) and T6 (right) for Neutral, Happy, and Angry stimulus conditions in OMOTENASHI and CONTROL.

Amplitude (μV)	OMOTENASHI		CONTROL	
	T5	T6	T5	T6
Neutral	− 13.1 ± 8.9	− 15.0 ± 8.5	− 13.1 ± 7.2	− 16.4 ± 8.2*
Happy	− 14.3 ± 8.2	− 16.8 ± 10.3	− 13.8 ± 9.0	− 16.2 ± 9.0
Angry	− 12.8 ± 9.9	− 15.1 ± 10.1	− 13.7 ± 9.1	− 16.7 ± 9.5

Data are presented as the mean and standard deviation for each group.

*p < 0.05: comparison with T5.

#### Peak latency of N170

The effects of *stimulus condition* (F = 1.460, p > 0.05, partial η^2^ = 0.037), *electrode* (F = 0.217, p > 0.05, partial η^2^ = 0.006)*,* and *subject group* (F = 0.153, p > 0.05, partial η^2^ = 0.004) on the peak latency of N170 were not significant, nor were any of the interactions between the parameters.

#### Maximum amplitude of N170

The *electrode* (F = 5.192, p < 0.05, partial η^2^ = 0.120) had a significant effect on the maximum amplitude of N170 (baseline-to-peak). The maximum amplitude of N170 was significantly larger at T6 (right) than at T5 (left). However, the effects of *stimulus condition* (F = 1.071, p > 0.05, partial η^2^ = 0.027) and *subject group* (F = 0.034, p > 0.05, partial η^2^ = 0.001) on the maximum amplitude of N170 were not significant, nor were any of the interactions between the parameters.

We investigated the differences in the maximum amplitude of N170 among *electrode* in each age group for each stimulus condition using post hoc analysis. In CONTROL, the maximum amplitude of N170 was significantly larger for Neutral at the T6 electrode (right) than at the T5 electrode (left).

#### Topographical map of N170

The topographical maps of N170 when the N170 amplitude in grand-averaged waveforms was largest at the T6 electrode (right) obtained for each subject group for all stimulus conditions (Neutral, Happy, and Angry) are shown in Fig. 2. The amplitude at the bilateral temporal areas was lower for each condition in CONTROL than in OMOTENASHI.

## Discussion

This psychophysical study can be summarized as follows: (1) the score on the favor rating test was significantly lower in OMOTENASHI than in CONTROL, and (2) OMOTENASHI favored Neutral significantly less than CONTROL.

We developed this favor rating test for use in this study. In CONTROL, angry faces were perceived as unfavorable, neutral faces as neither favorable nor unfavorable, and happy faces as favorable in this study. Therefore, we considered this favor rating test to be useful and appropriate for evaluating emotional perception.

The lower score on the favor rating test for OMOTENASHI in this study suggested that workers at hot spring inns are more sensitive to the facial emotion of guests due to their hospitality expertise. In addition, the workers at hot spring inns did not prefer a neutral face in the favor rating test because a neutral face may not make them feel at ease. These significant differences revealed a greater sensitivity to facial emotion and higher sense of responsibility in workers at hot spring spa inns than in common people, probably due to hospitality training for a long period.

The ERP results can be summarized as follows: (1) the maximum amplitude of P100 was significantly larger for OMOTENASHI than for CONTROL, (2) the maximum amplitude of P100 was significantly larger for Neutral in OMOTENASHI than in CONTROL at the right occipital electrode, (3) the maximum amplitude of P100 was significantly larger for Angry in OMOTENASHI than in CONTROL at the bilateral occipital electrodes, and (4) the peak latency and maximum amplitude of N170 were not significantly different between OMOTENASHI and CONTROL, although the amplitude in the topographical map of N170 at the bilateral temporal areas was lower for each condition in CONTROL than in OMOTENASHI.

P100 and N170 are considered to reflect activities of the early and late stages of face perception, respectively. Therefore, a difference in the P100 and unchanged N170 in OMOTENASHI suggested that workers at hot spring spa inns noticed facial emotion of guests faster than common people because of their experience in calming guests for a short period. In addition, the P100 amplitude in OMOTENASHI was significantly larger than in CONTROL, particularly for an angry face. This suggested that workers at hot spring spa inns are more sensitive to the uncomfortable feelings of guests than common people and they consider how to manage guests due to their hospitality expertise. This sensitivity in face perception may be one of the most important factors of OMOTENASHI. A previous study presented evidence of cortical hypervigilance in social phobia, expressed as generally increased P100 amplitudes to emotional faces, regardless of facial expression^22^. In another study, migraine patients had significantly more negative ERPs between 100 and 180 ms for all three picture categories (positive, negative, and neutral), and generally heightened sensitivity to all complex pictorial stimuli^23^. In addition, a study designed to investigate the differences in cortical processing of facial stimuli when neutral faces were presented in a context that involved information about emotional valence and self-reference demonstrated that depressed patients have larger mean P100 amplitudes than healthy controls across conditions (self-related/physically threatening, self-related/socially threatening, self-related/neutral, other-related/physically threatening, other-related/socially threatening, other-related/neutral)^24^. Therefore, our study is consistent with these previous studies.

Previous studies about visual perception revealed that the P100 amplitude increases more for attended stimuli than for non-attended stimuli^6^, and that emotion perception is automatic and unconscious due to bottom-up processing by the amygdala^25–28^, and/or top-down processing by orbital and ventromedial prefrontal cortices^29–32^. Based on previous studies, we also considered the possibility that hospitality expertise increased attention to emotion by top-down and/or bottom-up processing. Furthermore, a previous study revealed that patients who underwent right temporal lobe resection (rTLR) including the amygdala have a higher P100 amplitude than controls^33^. Our study is partly consistent with this previous rTLR patient study.

There are a number of limitations in this study. First, the favor rating test was developed for this study. Based on the results in CONTROL, this favor rating test may be useful to evaluate the impression of faces. However, there is a possibility that this test cannot be used by others as it is an original design.

Second, the peak latency and maximum amplitude of N170 were not significantly different between OMOTENASHI and CONTROL at both right and left temporal electrodes in this study, although the amplitude in the topographical map of N170 at the bilateral temporal areas was lower for each emotion condition in CONTROL than in OMOTENASHI. In addition, there were no significant differences among emotion conditions at both right and left temporal electrodes in each subject group. Based on previous studies, there were no significant differences as mentioned above in N170 due to not only the variability among individual subjects, but also the following possibilities: (1) the difference in the reference in EEG recording^34^, (2) the differences in the electrodes in N170 analysis^35–38^, (3) the difference in bandpass filter, especially the high-pass filter^39,40^, (4) the difference in the ISI^13^, (5) difference in face naturalness^15^, (6) the differences in figural facial expression features^16^, and (7) the differences in configural information of faces^17^. Therefore, further studies are warranted to eliminate these possibilities and confirm our findings.

Third, topographic differences suggest differences in neural generators because topography was a conventional criterion for defining ERP components^41^, and we partially agreed with the usefulness of topographic differences revealing whether two scalp topographies differ (e.g., Angry/Neutral). On the other hand, there were no significant differences in the P100 and N170 maximum amplitudes between each condition in each group, and we also confirmed that the subtraction method used in the topographic differences may show artificial activity and/or reducing real activity. Therefore, only the topographical maps of P100 and N170 were shown, and not the topographic differences. However, we did not deny that others did not easily recognize the differences between each condition in this study.

In conclusion, we investigated ERPs and psychophysical changes related to facial emotion perception based on Japanese hospitality (OMOTENASHI) expertise. The changes in P100 suggested that it is related to emotion perception and is affected by expertise in OMOTENASHI, differing from N170. Based on P100, workers at hot spring spa inns are more sensitive to the feelings of guests than common people due to their hospitality expertise. This is the first study to demonstrate the sensitivity in facial perception due to the specific type of hospitality OMOTENASHI.

## Methods and materials

### Subjects

Forty normal right-handed females with normal or corrected visual acuity participated in this study. The subjects were divided into two groups, i.e., OMOTENASHI (n = 21, age (mean and standard deviation): 50.5 ± 12.0 years), and CONTROL (n = 19, age: 50.4 ± 11.4 years). Subjects in OMOTENASHI were workers at hot spring spa inns in Gamagori city, Aichi Prefecture, Japan, which is a well-known hot spring spa area. On the other hand, subjects in CONTROL had no experience in the hospitality, service, medical, or education industry. There were no significant differences in age between OMOTENASHI and CONTROL by Welch's t test.

All subjects provided informed consent to participate in the experiment, which was approved by the ethics committee of the National Institute for Physiological Sciences. All experiments were conducted according to the Declaration of Helsinki. Each subject was compensated at the end of the experiment.

### Visual stimuli

**Figure 3:**
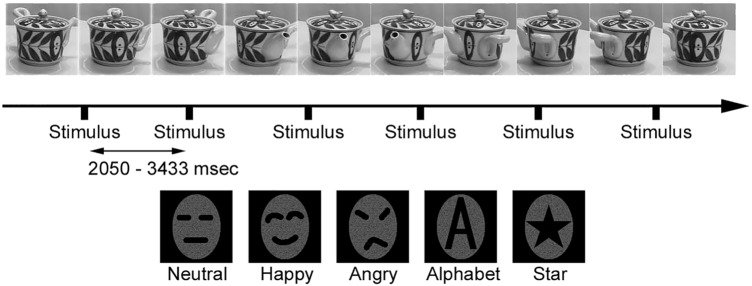
The background moving image of a ‘rotating teapot’ (top) and the examples of stimulus conditions (bottom) modified from our previous study^42^. The time of the inter-stimulus interval (ISI) was random within 2050–3433 ms. Examples of stimulus conditions. Neutral: neutral face stimuli, Happy: happy face stimuli, Angry: angry face stimuli, Alphabet: images of A, B, C, D, E, and F, Target: images of a star. The subjects were asked to push a button as quickly as possible when the target stimuli were presented. Images of face stimuli (Neutral, Happy, and Angry) in Fig. 3 were schematic faces made by the corresponding author, but real face images of Japanese (ATR Promotions, Inc., Kyoto, Japan), which had no hair, ears, or neck (3 male and 3 female faces), were used in the EEG recording and psychological tests, and the favor rating test of this study.

In the visual field, a rotating teapot with a rotation cycle of 4 s was projected as a background image, the same as the modified stimulus used in our previous study^42^ (Fig. 3).

The image of the teapot was in gray-scale. The total field of stimulation was 9.4 degrees (height) × 9.6 degrees (width), and the fixation point (red cross) was at the center of the visual field. Inserted into the background image were the following 5 stimuli (Fig. 3):

1. Neutral: neutral face stimuli.
2. Happy: happy face stimuli.
3. Angry: angry face stimuli.
4. Alphabet: images of A, B, C, D, E, and F.
5. Target: image of a star. The subjects were asked to push a button as quickly as possible when the target stimuli were presented.

In this stimulus presentation method, a rotating teapot continuously activated other visual areas except for the visual area related to face perception. Therefore, we thought this method may mainly evoke the responses in the visual areas related to the visual process of the face perception when face stimuli were presented, with minimizing and reducing the evoked responses in other visual areas.

We presented Alphabet and Target stimuli to ensure that the experimental task functioned as an implicit face perception task, and the luminance and contrast of the Alphabet stimuli differed from those of the neutral face (Neutral), happy face (Happy), and angry face (Angry) stimuli. Therefore, we analyzed the results obtained under conditions (1), (2), and (3).

We used neutral, happy, and angry faces of Japanese (ATR Promotions, Inc., Kyoto, Japan), which had no hair, ears, or neck (3 male and 3 female faces), and which were used in our previous study (Fig. 3)^21^, and subjects easily perceived and judged these stimuli as each emotion. Emotion rating tests were performed by ATR Promotions. Twenty-seven adults rated each face from 1 (low) to 7 (high) in each category of emotion (happy, sad, surprise, anger, disgust, fear, and contempt). As results of the test for the stimuli used in this study by ATR promotion, the happy face was highly rated as happy, the angry face was highly rated as angry, and the neutral face was rated as neither happy nor angry. In addition to the test by ATR Promotions, the test was performed after the experiments for all subjects who participated in this study. The results of this test in each group were the same as those in the test performed by ATR Promotions, and there were no significant differences between groups in any condition.

All face images were in gray-scale and unfamiliar to the subjects, and alphabet and star images were in gray-scale. The size of face, alphabet and star images was 9.4 degrees (height) × 9.6 degrees (width) the same as the teapot image (Fig. 3). The stimuli were shown for 500 ms. Each of the five conditions was automatically and randomly presented once or twice per one rotating circle of the teapot, and thus the time of the inter-stimulus interval (ISI) was random within 2050–3433 (2964.9 ± 186.9: means and standard deviation) ms.

A rotating teapot and visual stimuli were presented on a personal computer (OPTIPLEX 980: Dell Technologies, USA) and cathode ray tube (CRT) screen (FlexScan T961: Eizo Nanao Corporation, Japan). The distance between the subject’s eyes and the CRT screen was 110 cm. Stimuli were projected centrally in the visual presentation field. Subjects sat on the chair for EEG measurement and were asked to focus on a small red cross at the center of the stimulus during the experiment. To minimize habituation and drowsiness, each subject took part in more than 10 short-term recording sessions.

### Psychophysical study

Workers in the hospitality industry, such as at hot spring spa inns, scrutinize guests as to their favorability. Therefore, we made an original test scoring how favorable presented image of faces are, termed the favor rating test, and performed it after the experiment. Subjects rated each face from 1 (low: not favorite) to 7 (high: favorite) for Neutral, Happy, and Angry. For favor rating, a repeated measures two-way ANOVA was performed with *stimulus condition* (Neutral, Happy, or Angry) as a within-subject factor and *subject group* (OMOTENASHI, CONTROL) as a between-subject factor. In addition, Welch's t test was used between OMOTENASHI and CONTROL to investigate the effects of hospitality on each face and p < 0.05 was considered significant.

### ERP recording and data analysis

ERPs were recorded using 7 electrodes according to the international 10–20 system (Fz, Cz, T5, T6, Pz, O1, and O2), with an additional 2 electrodes, T5’ (2 cm below T5) and T6’ (2 cm below T6)^21,43^ with Ag/AgCl disk electrodes. The impedance of electrodes was kept below 5 k ohm. The reference was placed at the tip of the nose far from the temporal area because the reference used in this study may be less affected by the temporal area activity than other references, and useful for recording N170 judging from our previous studies^21,44^. Electrooculography (EOG) was also recorded using an electrode located above the right eye and the reference electrode to assess blinking and eye movements.

We used Neurofax (Nihon-Kohden, Tokyo, Japan) to record the data and EPlyzer (Kiseei Comtec, Nagano, Japan) to analyze the data. The bandpass filter was 0.03–120 Hz and the sampling rate was 1000 Hz during on-line recording. Eye movements of subjects were monitored during EEG recordings with EOG. Epochs in which signal variations of EEG and EOG were larger than ± 150 µV were automatically excluded from the averaging.

As for the ERP analysis after on-line recording, the finite impulse response bandpass filter with filter properties forward was 1–30 Hz off-line after stimulus onset to minimize the influence of artifacts, and we confirmed that this bandpass filter did not smear the components or alter the amplitude of the components for each subject. We checked each epoch for each stimulus in each subject off-line, and epochs in signal variations of EEG and EOG that were large were excluded from the off-line averaging by visual inspection. More than 40 epochs were averaged off-line for each condition in each subject. The time window for the analysis ran from 100 ms before to 400 ms, and the data obtained during the 100 ms before stimulus onset were used as the baseline.

The peak latency and maximum amplitude (baseline-to-peak) of the P100 and N170 components were measured at the O1 (left) and O2 (right), and at the T5 (left) and T6 (right) electrodes respectively. The peak latency when the amplitude of the P100 and N170 components was maximal after stimulus onset for each condition, and the maximum amplitude of the component were individually measured visually within the range of 85–175 ms for P100 and 145–280 ms for N170. In addition, the distribution of P100 and N170 on the scalp was shown by the topographical maps of P100 and N170, when P100 amplitude in grand-averaged waveforms was largest at the O2 electrode in each subject group for all stimulus conditions (Neutral, Happy, and Angry), and when N170 in grand-averaged waveforms was largest at the T6 electrode in each subject group for all stimulus conditions (Neutral, Happy, and Angry)^41^.

For peak latency and maximum amplitude of P100 and N170, a repeated measures three-way ANOVA was performed with *stimulus condition* (Neutral, Happy, or Angry) and *electrode* (O1, O2 for the P100, and T5, T6 for the N170) as within-subject factors, and *subject group* (OMOTENASHI and CONTROL) as a between-subject factor to examine the effects of hospitality. Lastly, a repeated measures two-way factorial ANOVA was performed with *stimulus condition* (Neutral, Happy, or Angry) and *electrode* as factors in OMOTENASHI or CONTROL to examine the effects of condition and electrode in each subject group. Welch's t test was then performed with *subject group* (OMOTENASHI and CONTROL) for each stimulus (Neutral, Happy, or Angry) at each electrode.

The degrees of freedom were corrected using the Greenhouse–Geisser correction coefficient epsilon, and F and p values were recalculated. The Bonferroni multiple comparison test was used for the post hoc analysis and p < 0.05 was considered significant. In addition, Welch's t test was used between OMOTENASHI and CONTROL for peak latency and maximum amplitude, and p < 0.05 was considered significant.

## Data Availability

The data that support the findings of this study are available from the corresponding author, K.M., upon reasonable request.
